# Association between ultra-processed food intake and osteoarthritis prevalence among U.S. adults: A cross-sectional study using NHANES data

**DOI:** 10.1097/MD.0000000000051343

**Published:** 2026-10-30

**Authors:** Hongming Liang, Hongchun Peng, Li Shen, Xin Liu, Cheng Li, Liwen Xue

**Affiliations:** a Changsha Central Hospital, Changsha, Hunan, China; b Xiangya Second Hospital of Central South University, Changsha, Hunan, China.

**Keywords:** diet, NHANES, osteoarthritis, ultra-processed foods

## Abstract

This study examines the association between ultra-processed food (UPF) intake and osteoarthritis (OA) prevalence among U.S. adults aged 40 years and older. Data for this study were from the U.S. National Health and Nutrition Examination Survey between 2015 and 2020. Multivariable logistic regression models, adjusted for sociodemographic, lifestyle, and clinical factors, were employed to assess the association between UPF intake (categorized into quartiles using the NOVA classification) and OA prevalence. Restricted cubic spline analysis evaluated the dose-response relationship, and machine learning models were applied for predictive modeling. The final analysis included a weighted sample representing approximately 216,413,969 U.S. adults aged ≥40 years. Fully adjusted multivariable logistic regression revealed a positive association between higher UPF intake quartiles and OA prevalence (adjusted odds ratio [aOR] for highest vs lowest quartile = 1.24, 95% confidence interval: 1.05–1.48, *P* = .01). Restricted cubic spline analysis indicated a linear dose-response relationship (*P* for linearity = .02). Subgroup analysis identified a statistically significant interaction by body mass index category (*P* for interaction = .033), with a significant association in the obesity subgroup (odds ratio = 1.16, 95% confidence interval: 1.05–1.30; *P* = .006). Among machine learning models, XGBoost demonstrated superior predictive accuracy (area under the receiver operating characteristic curve = 0.96). Shapley Additive Explanation analysis identified age, body mass index, and UPF intake as the most influential predictors. Higher UPF consumption was associated with higher OA prevalence. Prospective studies are needed to examine temporal relationships and causal mechanisms.

## 1. Introduction

Osteoarthritis (OA) is a multifactorial whole-joint disorder characterized by cartilage degeneration, synovial inflammation, and metabolic dysregulation.^[1]^ Increasing evidence suggests that obesity-related inflammation and metabolic disturbances may contribute to OA progression.^[2]^ Early, macrophage-rich synovitis and angiogenic remodeling of the synovial lining release cytokines (e.g., tumor necrosis factor alpha (TNF-α), interleukin-1 (IL-1), and interleukin-6 (IL-6)) that tilt chondrocytes toward catabolism via matrix metalloproteinase-13 (MMP-13) and a disintegrin and metalloproteinase with thrombospondin motifs 5 (ADAMTS-5), while subchondral bone exhibits porosity changes and aberrant remodeling that propagate cartilage damage through bone–cartilage crosstalk.^[1,3]^ Aging-related mitochondrial dysfunction, oxidative stress, and cellular senescence amplify these responses and blunt repair; in parallel, obesity-related adipokines (leptin, resistin, visfatin, others) add a systemic inflammatory tone that accelerates matrix breakdown.^[1]^ Innate immune sensors: including Toll-like receptor 4 and the NOD-like receptor protein 3 inflammasome (NLRP3) inflammasome: are increasingly implicated in knee OA synovitis, where their activation triggers nuclear factor kappa B (NF-κB) signaling and IL-1β maturation, reinforcing a self-perpetuating inflammatory circuit.^[4]^ In addition, advanced glycation end products (AGEs) accumulate with aging and hyperglycemia to cross-link cartilage collagen and engage receptor for AGEs receptor for advanced glycation end products (RAGE) signaling, promoting chondrocyte apoptosis, suppression of autophagy, and matrix stiffening.^[5]^ A gut–joint axis has also emerged: human and animal studies link intestinal dysbiosis to increased permeability and systemic translocation of microbial products such as lipopolysaccharide (LPS), which activate synovial and chondrocyte receptors and associate with OA severity.^[6,7]^

Ultra-processed foods (UPFs): industrial formulations defined by the NOVA system and characterized by fractionated ingredients and cosmetic additives: now account for a large share of energy intake in many settings.^[8]^ In an inpatient randomized trial, ad libitum UPF diets increased energy intake and body weight within 2 weeks compared with minimally processed diets matched for macronutrients, sugars, sodium, and fiber, indicating that processing itself (for example, texture, eating rate, hyper-palatability) promotes overconsumption.^[9]^ An umbrella review of meta-analyses further associates UPF exposure with broad adverse health outcomes, especially cardiometabolic disease and mortality.^[10]^ Several UPF-specific features plausibly intersect with OA biology. First, common emulsifiers (for example, carboxymethylcellulose and polysorbate-80) erode the intestinal mucus layer and increase microbiota encroachment and permeability in animals^[11]^; in a controlled human trial, carboxymethylcellulose altered microbiota composition, reduced short-chain fatty acids, and induced mucus encroachment in susceptible participants, changes consistent with low-grade inflammation and metabolic disruption.^[12]^ These perturbations could raise systemic LPS levels and engage toll-like receptor 4/NLRP3 pathways already implicated in OA synovitis.^[4,13]^ Second, UPF processing can increase dietary AGE load, potentially augmenting tissue AGE burden and RAGE signaling that stiffen cartilage and amplify catabolic responses.^[5,14]^ Third, UPF-driven positive energy balance promotes obesity and visceral adiposity, thereby intensifying joint loading and adipokine-driven inflammation linked to OA progression.^[1,9,15]^ Consequently, elucidating UPF’s contribution to OA pathogenesis could redefine preventive strategies.

This study addresses these gaps through a cross-sectional analysis of 2015 to 2020 National Health and Nutrition Examination Survey (NHANES) data, representing a weighted sample of 216,413,969 U.S. adults aged ≥40 years. By integrating multivariable logistic regression with restricted cubic splines (RCS) and complementary machine learning approaches, we further explored whether UPF intake contributes additional predictive information beyond conventional risk factors. Complementary machine learning modeling confirmed superior predictive accuracy (XGBoost area under the receiver operating characteristic curve [AUROC] = 0.96), with SHapley Additive exPlanations (SHAP) interpretation underscoring UPF intake, age, and body mass index (BMI) as dominant risk factors. Collectively, these findings highlight the potential relevance of UPF intake as a dietary factor associated with OA prevalence.

## 2. Materials and methods

### 2.1. Study population

This cross-sectional study was conducted using data from the NHANES, a continuous, nationally representative survey designed to assess the health and nutritional status of the U.S. population.^[16]^ Data from the 2015 to 2020 NHANES cycles were included based on the availability of dietary and health-related variables relevant to the study. A total of 25,531 participants were initially identified from the NHANES 2015 to 2020 cycles. Participants were eligible for inclusion if they were aged ≥40 years and had available dietary intake data and OA assessment. This age threshold was selected because OA prevalence increases substantially among older adults. Participants with missing UPF intake data or missing OA assessment were excluded. After applying the eligibility criteria, the remaining participants were included in the final analytical sample. The unweighted participant sequence was 25,531 in the combined files, 14,951 aged 40 years or older, 14,018 with a reliable Day 1 dietary recall and dietary weight, 12,723 with a known OA outcome and nonmissing UPF exposure, and 12,307 complete cases for the primary model. Detailed participant selection is provided in Supplemental Digital Content 1, Table S1, Supplemental Digital Content 1.

The data used in this study are publicly available from the NHANES website maintained by the Centers for Disease Control and Prevention (CDC). All datasets from the 2015 to 2020 survey cycles can be accessed at https://www.cdc.gov/nchs/nhanes. NHANES data collection was approved by the National Center for Health Statistics Research Ethics Review Board, and all participants provided informed consent. This secondary analysis used de-identified public data and required no additional local ethics review.

### 2.2. Assessment of UPF intake

Dietary intake data were obtained from the 24-hour dietary recall component of NHANES. UPF intake was estimated based on the NOVA classification system, which categorizes foods according to the extent and purpose of food processing. According to the NOVA framework, UPFs are industrial formulations made from substances extracted from foods or synthesized from food constituents, typically containing additives used to enhance flavor, texture, or shelf life. This classification approach has been widely applied in nutritional epidemiological studies to evaluate the health effects of food processing.^[8]^ Because NHANES does not provide an official NOVA classification or complete brand-level ingredient information, classification used prespecified food-category and description rules. Records requiring description review were retained with a review flag; residual exposure misclassification cannot be excluded. The classification-rule audit and uncertainty summaries are provided in Supplemental Digital Content 4 to 6 (Tables S4a, Supplemental Digital Content 8, S4b, Supplemental Digital Content 2, and S5, Supplemental Digital Content 9), with the underlying audit data in Supplemental Digital Content 11 and 12 (Files 1, Supplemental Digital Content 3 and 2, Supplemental Digital Content 12).

Individual food items from the NHANES dietary recall database were classified according to the NOVA criteria. The total amount of UPF consumed per day was calculated by summing the gram amounts of all UPF items reported by each participant, and the corresponding energy intake from UPFs was calculated based on the caloric contribution of these foods. UPF intake was analyzed continuously and in quartiles: Q1 <=406.4 kcal/day, Q2 > 406.4 to <=750.4 kcal/day, Q3 > 750.4 to <=1094.3 kcal/day, and Q4 > 1094.3 kcal/day. Absolute UPF intake was selected as the primary exposure metric because it directly reflects the actual quantity of UPFs consumed by participants and provides an intuitive measure of individual exposure. Although energy-adjusted or weight-adjusted UPF metrics may capture different aspects of dietary exposure, absolute intake was chosen to evaluate the association between the amount of UPF consumption and OA prevalence.

Sensitivity analyses modeled absolute UPF intake per 100 g/day and UPF energy and food-weight proportions per 10-percentage-point increment.

### 2.3. Outcome definition

The outcome of interest was the presence of osteoarthritis, determined via self-report from the NHANES Medical Conditions Questionnaire. Participants who answered “Yes” to the question “Has a doctor or other health professional ever told you that you had arthritis?” (MCQ160A), and subsequently identified the type as “OA” in response to MCQ195, were classified as having OA. Participants with unknown outcome responses were excluded. The OA response-path audit is provided in Supplemental Digital Content 3, Table S3, Supplemental Digital Content 4.

### 2.4. Covariates

Covariates were selected based on previous literature and established risk factors associated with osteoarthritis and dietary exposures. Demographic covariates included age (continuous), sex (male/female), race/ethnicity (Non-Hispanic White, Non-Hispanic Black, Hispanic, Other), education level (less than high school, high school, more than high school), poverty-income ratio (PIR), analyzed both as a continuous variable and as a categorical variable (<1.30, 1.30–3.49, and ≥3.50), and marital status (living alone/living with a partner). PIR was modeled continuously; the categories <1.30, 1.30 to 3.49, and >=3.50 were used for descriptive reporting. Lifestyle factors included smoking status (never, former, current), alcohol use (yes/no), and physical activity (PA) level. The source variables and analytical coding are listed in Supplemental Digital Content 2, Table S2, Supplemental Digital Content 5.

Metabolic health variables, including BMI, diabetes, hypertension, and dyslipidemia, were additionally included in the fully adjusted model because they are well-established risk factors for osteoarthritis and may influence the observed association between UPF intake and OA prevalence. However, we acknowledge that some metabolic factors may also represent potential intermediate variables in the pathway linking UPF consumption to OA. Therefore, adjustment for these variables was intended to evaluate the association between UPF intake and OA independent of metabolic comorbidities rather than estimate the total causal effect of UPF exposure.

### 2.5. Statistical analysis

All analyses incorporated NHANES survey weights, strata, and primary sampling units to account for the complex, multistage probability sampling design and to produce nationally representative estimates. For the combined 5.2-year period, Day 1 dietary weights were calculated as WTDRD1 × 2/5.2 for 2015 to 2016 and WTDRD1PP × 3.2/5.2 for 2017-March 2020. The variables SDMVSTRA and SDMVPSU were used as strata and primary sampling units, respectively. Descriptive statistics were used to compare baseline characteristics between OA and non-OA participants, using Student’s *t* tests for continuous variables and chi-square tests for categorical variables. PIR was analyzed continuously to preserve the full information of the variable and categorically to facilitate interpretation of differences across socioeconomic groups. Multivariable logistic regression models were employed to assess the association between UPF intake and OA prevalence. Three models were developed: Model 1 was unadjusted; Model 2 adjusted for gender, age, race, education, and marital status; and Model 3 further adjusted for smoking behavior, alcohol consumption, diabetes, BMI, PIR, hypertension, dyslipidemia, and PA. Results were expressed as odds ratios (ORs) with 95% confidence intervals (CIs). Model calibration was assessed using design-based decile calibration and the weighted Brier score.

To explore the dose-response relationship between UPF intake and OA-related outcomes, RCS regression models were constructed using the original log-transformed UPF scale, with a reference value of 3.23 on that scale. Exploratory subgroup analyses were conducted to assess whether the association between UPF intake and OA prevalence differed across selected demographic and clinical characteristics, including sex, BMI category (<25, 25–29.9, ≥30 kg/m^2^), hypertension status, and diabetes status. Interaction terms were tested on the multiplicative scale by including cross-product terms between UPF intake and each stratifying variable in the fully adjusted logistic regression models. All statistical analyses were performed using R software (version 4.4.3) and a two-sided *P*-value <.05 was considered statistically significant.

### 2.6. Machine learning analysis

To complement traditional epidemiological analyses, machine learning models were applied as exploratory predictive analyses to evaluate the contribution of UPF intake within a multivariable risk prediction framework. These models were not intended to establish causal relationships or replace regression-based inference.

First, the least absolute shrinkage and selection operator (LASSO) logistic regression was employed for feature selection. Ten-fold cross-validation was applied to identify the optimal penalization parameter (lambda), and variables with nonzero coefficients at the minimum lambda value were retained for further modeling.

Subsequently, 6 supervised learning algorithms were trained using the selected features: eXtreme Gradient Boosting (XGBoost), Light Gradient Boosting Machine (LightGBM), Categorical Boosting (CatBoost), Gradient Boosting Decision Tree (GBDT), Random Forest, and Naïve Bayes (NB). The dataset was randomly split into a training set (70%) and a testing set (30%). Model performance was evaluated using accuracy, recall, precision, F1-score, Matthews correlation coefficient, AUROC, and calibration error. Receiver operating characteristic curve and calibration curves were generated to assess model discrimination and probability calibration, respectively. Among the 6 models, XGBoost exhibited the highest predictive performance and was selected as the final model. To validate its robustness, 5-fold cross-validation was conducted, and the average AUROC and calibration performance were reported across folds.

Finally, SHAP were applied to interpret the contribution of each predictor to the model’s output. Feature importance rankings and SHAP beeswarm plots were used to visualize the magnitude and direction of variable influence on individual predictions, thereby enhancing model interpretability and transparency. As an additional internal analysis, we compared a conventional prediction model with the corresponding model including UPF in a 70:30 training-test split. This comparison was intended to quantify incremental discrimination and was not used to replace the 6-model evaluation.

## 3. Results

### 3.1. Baseline characteristics of participants

The final analysis represented an estimated population of 216,413,969 U.S. adults aged 40 years and older after applying NHANES sampling weights (Table 1). Among them, 30,089,267 individuals (13.91%) self-reported a diagnosis of OA. The weighted mean age of the total population was 48.24 years (SD 17.23), with OA participants being substantially older (62.43 ± 12.60 years) than those without OA (45.95 ± 16.77 years, *P* < .001). The majority of OA patients were female (66.60%), compared to 49.11% in the non-OA group (*P* < .001). The final unweighted analytic sample included 12,307 participants, including 1524 participants with OA. Detailed survey-weighted baseline characteristics are presented in Table 1.

**Table 1:** Weighted baseline characteristics of the study population.

Characteristic	N	Overall N = 216,413,969	OA		*P*-value
			No N = 186,324,702	Yes N = 30,089,267	
Age	216,413,969	48.24 (17.23)	45.95 (16.77)	62.43 (12.60)	<.001
PIR	216,413,969	3.07 (1.56)	3.04 (1.57)	3.22 (1.53)	.030
BMI	216,413,969	29.70 (7.14)	29.46 (7.04)	31.21 (7.54)	<.001
HDL	216,413,969	54.40 (16.70)	53.90 (16.11)	57.50 (19.67)	<.001
HsCRP	216,413,969	3.84 (6.87)	3.77 (6.45)	4.29 (9.01)	<.001
TC	216,413,969	189.16 (40.41)	188.50 (40.26)	193.20 (41.09)	.003
Ultra-processed food (grams), mean (sd)	216,413,969	788.75 (508.57)	799.53 (515.31)	722.05 (459.22)	<.001
Ultra-processed food (kcal), mean (sd)	216,413,969	803.23 (509.42)	809.96 (511.23)	761.61 (496.24)	.033
Gender, n (p%)	216,413,969				<.001
Male		104,862,571 (48.45%)	94,813,259 (50.89%)	10,049,312 (33.40%)	
Female		111,551,397 (51.55%)	91,511,443 (49.11%)	20,039,954 (66.60%)	
Race, n (p%)	216,413,969				<.001
Mexican American		17,812,767 (8.23%)	16,983,085 (9.11%)	829,681 (2.76%)	
Non-Hispanic Black		14,780,009 (6.83%)	13,780,662 (7.40%)	999,348 (3.32%)	
Non-Hispanic White		140,915,490 (65.11%)	116,828,127 (62.70%)	24,087,363 (80.05%)	
Other Hispanic		23,951,551 (11.07%)	21,824,269 (11.71%)	2127,282 (7.07%)	
Other race		18,954,152 (8.76%)	16,908,559 (9.07%)	2045,592 (6.80%)	
Education, n (p%)	216,413,969				.499
Above high school		16,106,541 (7.44%)	14,149,973 (7.59%)	1956,569 (6.50%)	
High school		53,501,021 (24.72%)	45,692,309 (24.52%)	7808,712 (25.95%)	
Under high school		146,806,406 (67.84%)	126,482,420 (67.88%)	20,323,986 (67.55%)	
Marital, n (p%)	216,413,969				.136
Living alone		128,350,438 (59.31%)	109,735,353 (58.89%)	18,615,084 (61.87%)	
Living with a partner		88,063,531 (40.69%)	76,589,349 (41.11%)	11,474,182 (38.13%)	
PIR_Group, n (p%)	216,413,969				.016
<1.30		25,338,509 (11.71%)	22,644,839 (12.15%)	2693,669 (8.95%)	
1.30~3.49		113,639,365 (52.51%)	98,075,084 (52.64%)	15,564,281 (51.73%)	
≥3.50		77,436,095 (35.78%)	65,604,779 (35.21%)	11,831,316 (39.32%)	
BMI_Group, n (p%)	216,413,969				<.001
Normal		57,582,126 (26.61%)	51,651,897 (27.72%)	5930,229 (19.71%)	
Obese		69,516,404 (32.12%)	60,197,788 (32.31%)	9318,616 (30.97%)	
Overweight		89,315,439 (41.27%)	74,475,017 (39.97%)	14,840,422 (49.32%)	
Hypertension, n (p%)	216,413,969				<.001
No		70,583,383 (32.61%)	53,693,523 (28.82%)	16,889,860 (56.13%)	
Yes		145,830,586 (67.39%)	132,631,179 (71.18%)	13,199,406 (43.87%)	
Dyslipidemia, n (p%)	216,413,969				<.001
No		74,868,571 (34.60%)	57,105,157 (30.65%)	17,763,413 (59.04%)	
Yes		141,545,398 (65.40%)	129,219,545 (69.35%)	12,325,853 (40.96%)	
Diabetes, n (p%)	216,413,969				<.001
No		25,431,587 (11.75%)	19,447,796 (10.44%)	5983,791 (19.89%)	
Yes		190,982,381 (88.25%)	166,876,906 (89.56%)	24,105,475 (80.11%)	
PA, n (p%)	216,413,969				.016
No		102,637,199 (47.43%)	89,971,733 (48.29%)	12,665,466 (42.09%)	
Yes		113,776,770 (52.57%)	96,352,969 (51.71%)	17,423,800 (57.91%)	
Smoke, n (p%)	216,413,969				<.001
No		93,683,299 (43.29%)	78,074,304 (41.90%)	15,608,995 (51.88%)	
Yes		122,730,670 (56.71%)	108,250,398 (58.10%)	14,480,272 (48.12%)	
Drink, n (p%)	216,413,969				.041
No		78,032,614 (36.06%)	66,002,282 (35.42%)	12,030,332 (39.98%)	
Yes		138,381,355 (63.94%)	120,322,420 (64.58%)	18,058,935 (60.02%)	

BMI = body mass index, HDL = high-density lipoprotein cholesterol, HsCRP = high-sensitivity C-reactive protein, OA = osteoarthritis, PA = physical activity, PIR = poverty-income ratio, TC = total cholesterol.

OA prevalence varied significantly across racial and ethnic groups. Non-Hispanic White individuals accounted for 80.05% of the OA group, whereas Mexican Americans and Non-Hispanic Black participants were underrepresented (2.76% and 3.32%, respectively, *P* < .001). Regarding socioeconomic status, individuals with OA had a higher mean PIR (3.22 ± 1.53) compared to those without OA (3.04 ± 1.57, *P* = .030), though the categorical PIR distribution showed that a lower proportion of OA patients were from the lowest income group (<1.30, 8.95% vs 12.15%, *P* = .016). With respect to education, no significant differences were observed between groups (*P* = .499). Marital status also did not differ substantially (*P* = .136), although a higher proportion of OA patients lived alone (61.87% vs 58.89%). Obesity was more common in the non-OA group (32.31%) than in the OA group (30.97%), and OA participants were more likely to be overweight (49.32% vs 39.97%, *P* < .001). The OA group also had significantly higher BMI (31.21 ± 7.54 vs 29.46 ± 7.04, *P* < .001), higher hs-C-reactive protein (4.29 ± 9.01 vs 3.77 ± 6.45 mg/L, *P* < .001), and higher HDL cholesterol (57.50 ± 19.67 vs 53.90 ± 16.11 mg/dL, *P* < .001). In terms of comorbidities, non-OA patients had significantly higher rates of hypertension (71.18% vs 43.87%), dyslipidemia (69.35% vs 40.96%), and diabetes (89.56% vs 80.11%) compared to OA participants (all *P* < .001). Notably, OA participants reported lower mean UPF intake (722.05 ± 459.22 grams vs 799.53 ± 515.31 grams, *P* < .001) and lower energy intake from UPFs (761.61 ± 496.24 kcal vs 809.96 ± 511.23 kcal, *P* = .033). In terms of lifestyle factors, a greater proportion of OA participants reported engaging in PA (57.91% vs 51.71%, *P* = .016), while fewer reported smoking (48.12% vs 58.10%, *P* < .001). The prevalence of alcohol consumption was slightly lower in the OA group (60.02% vs 64.58%, *P* = .041).

### 3.2. Association between UPF intake and osteoarthritis prevalence

The association between UPF intake and the prevalence of osteoarthritis was examined using multivariable logistic regression models, and results are presented in Table 2. In the unadjusted model (Model 1), higher total UPF intake was significantly associated with lower odds of OA (OR = 0.9193, 95% CI: 0.8705–0.9707, *P* = .0025). However, after adjusting for demographic variables (Model 2), the direction of the association reversed (OR = 1.1027, 95% CI: 1.0378–1.1715, *P* = .0016), and remained statistically significant in the fully adjusted model (Model 3: OR = 1.0935, 95% CI: 1.0284–1.1627, *P* = .0043), suggesting a possible confounding effect.

**Table 2:** Association between UPF intake and osteoarthritis prevalence.

Variable	OR (95% CI), *P*-value		
	Model 1	Model 2	Model 3
UPF	0.9193 (0.8705, 0.9707) 0.0025	1.1027 (1.0378, 1.1715) 0.0016	1.0935 (1.0284, 1.1627) 0.0043
Stratified by UPF quartiles			
Q1 (≤406.4 kcal/day)	Reference	Reference	Reference
Q2 (>406.4–≤ 750.4 kcal/day)	1.0874 (0.9392, 1.2590) 0.2625	1.1602 (0.9910, 1.3582) 0.0647	1.1636 (0.9918, 1.3651) 0.0630
Q3 (>750.4–≤ 1094.3 kcal/day)	0.8753 (0.7514, 1.0197) 0.0873	1.2411 (1.0495, 1.4677) 0.0116	1.2015 (1.0132, 1.4248) 0.0347
Q4 (>1094.3 kcal/day)	0.8356 (0.7163, 0.9748) 0.0223	1.2749 (1.0763, 1.5103) 0.0049	1.2435 (1.0480, 1.4753) 0.0125
*P* for trend	.0021	.0032	.0119

Model 1 was unadjusted. Model 2 adjusted for age, sex, race/ethnicity, education, and marital status. Model 3 additionally adjusted for PIR, smoking status, alcohol use, physical activity, BMI, diabetes, hypertension, and dyslipidemia.

BMI = body mass index, CI = confidence interval, OR = odds ratio, PIR = poverty-income ratio, Q = quartile, UPF = ultra-processed food.

When UPF intake was categorized into quartiles, a positive association between higher quartiles and OA prevalence was observed in both adjusted models. Compared with the lowest quartile (Q1, reference group), participants in the highest quartile (Q4) had significantly higher odds of OA in Model 2 (OR = 1.2749, 95% CI: 1.0763–1.5103, *P* = .0049) and Model 3 (OR = 1.2435, 95% CI: 1.0480–1.4753, *P* = .0125). The associations were also significant for the third quartile (Q3 vs Q1) in both models (Model 2: OR = 1.2411, 95% CI: 1.0495–1.4677, *P* = .0116; Model 3: OR = 1.2015, 95% CI: 1.0132–1.4248, *P* = .0347). No significant associations were found for the second quartile (Q2) in any model (*P* > .05). A significant linear trend across quartiles was observed (Model 3: *P* for trend = .0119). In sensitivity analyses, UPF energy percentage was associated with OA prevalence per 10-percentage-point increment (OR = 1.20, 95% CI: 1.02–1.40; *P* = .025), as was UPF food-weight percentage (OR = 1.20, 95% CI: 1.04–1.40; *P* = .017). Absolute UPF intake per 100 g/day was not statistically significant (OR = 1.01, 95% CI: 0.99–1.03; *P* = .141). For the fully adjusted model, the design-based decile calibration *P* value was .384, the weighted Brier score was 0.103 (95% CI: 0.094–0.112), and Cox-Snell and Nagelkerke pseudo-R2 values were 0.131 and 0.236, respectively. Detailed survey-weighted association estimates are presented in Table 2; sensitivity and model-fit results are provided in Supplemental Digital Content 7 and 8 (Tables S6 and S7, Supplemental Digital Content 6).

### 3.3. Dose-response relationship between UPF intake and osteoarthritis prevalence

RCS regression was conducted using the original log-transformed UPF scale to explore the dose-response relationship between dietary UPF intake and the odds of osteoarthritis. As shown in Figure 1, the RCS curve suggested a generally positive association between UPF intake and OA prevalence. The test for overall association was statistically significant (*P* = .021), indicating a meaningful relationship between the exposure and outcome. However, the test for nonlinearity was not significant (*P* = .29), suggesting that the association follows a linear pattern rather than a curved or threshold-dependent shape.

**Figure 1.**
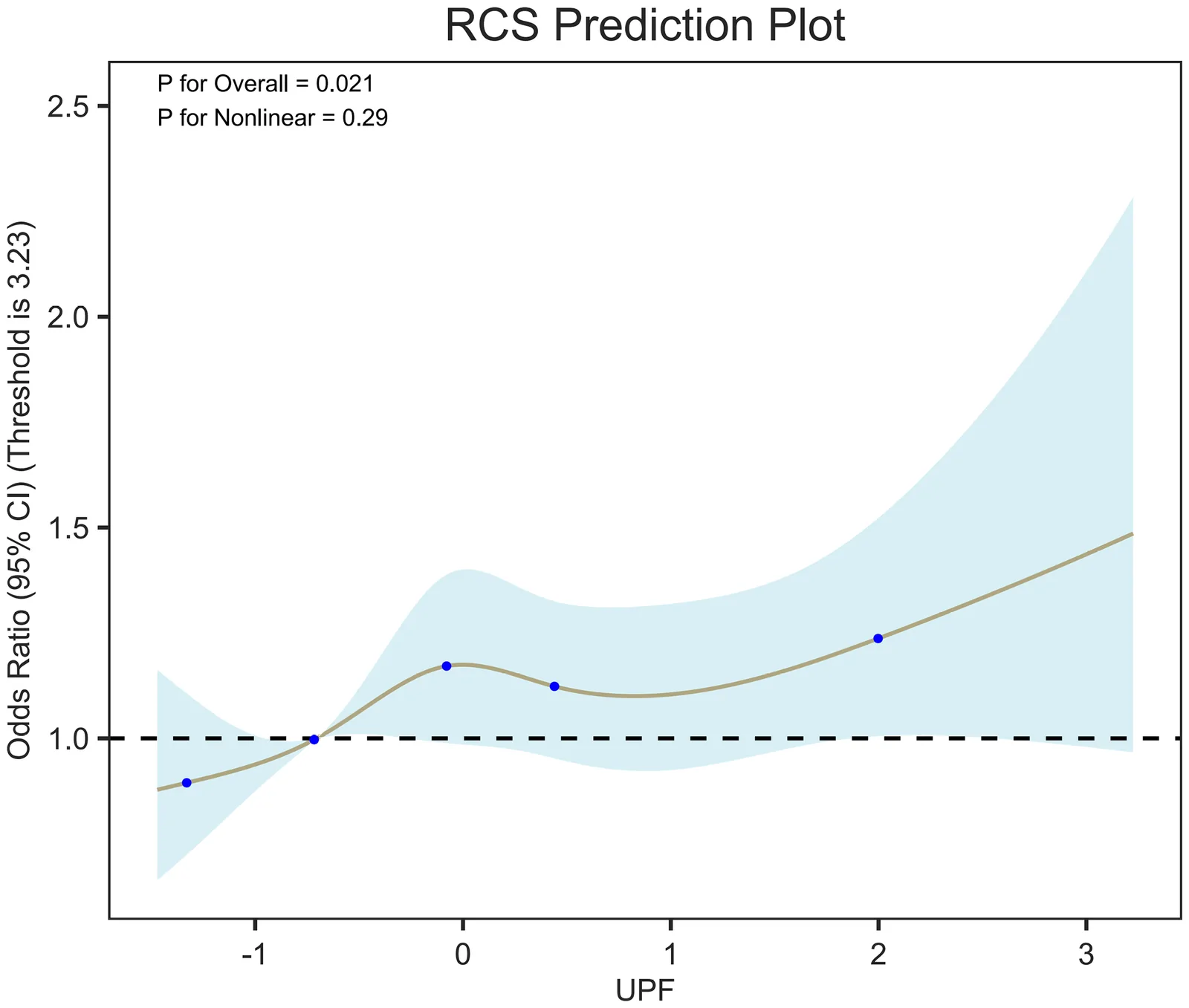
Dose-response relationship between ultra-processed food intake and osteoarthritis prevalence. The restricted cubic spline plot illustrates the association between continuous ultra-processed food (UPF) intake and the adjusted odds ratio (OR) for osteoarthritis. The solid line represents the estimated OR, and the shaded area represents the 95% confidence interval. The reference point (OR = 1) was set at the median value of log-transformed UPF intake (3.23). The analysis indicates a significant linear increase in osteoarthritis odds with higher UPF intake (*P* for overall association = .021; *P* for nonlinearity = .29). CI = confidence interval, RCS = restricted cubic spline.

The risk of OA appeared to increase progressively with higher levels of UPF intake, relative to the reference value (3.23) on the transformed scale. These findings are consistent with the logistic regression results and further support a monotonic increase in OA prevalence with increasing intake levels, rather than a U-shaped or J-shaped relationship.

### 3.4. Subgroup analyses

To further explore potential effect modifiers, subgroup analyses were conducted across key demographic, lifestyle, and health-related variables, including PIR, BMI, hypertension, diabetes, PA, smoking, alcohol consumption, dyslipidemia, and gender (Fig. 2). The association between UPF intake and osteoarthritis prevalence was generally consistent across most subgroups.

**Figure 2.**
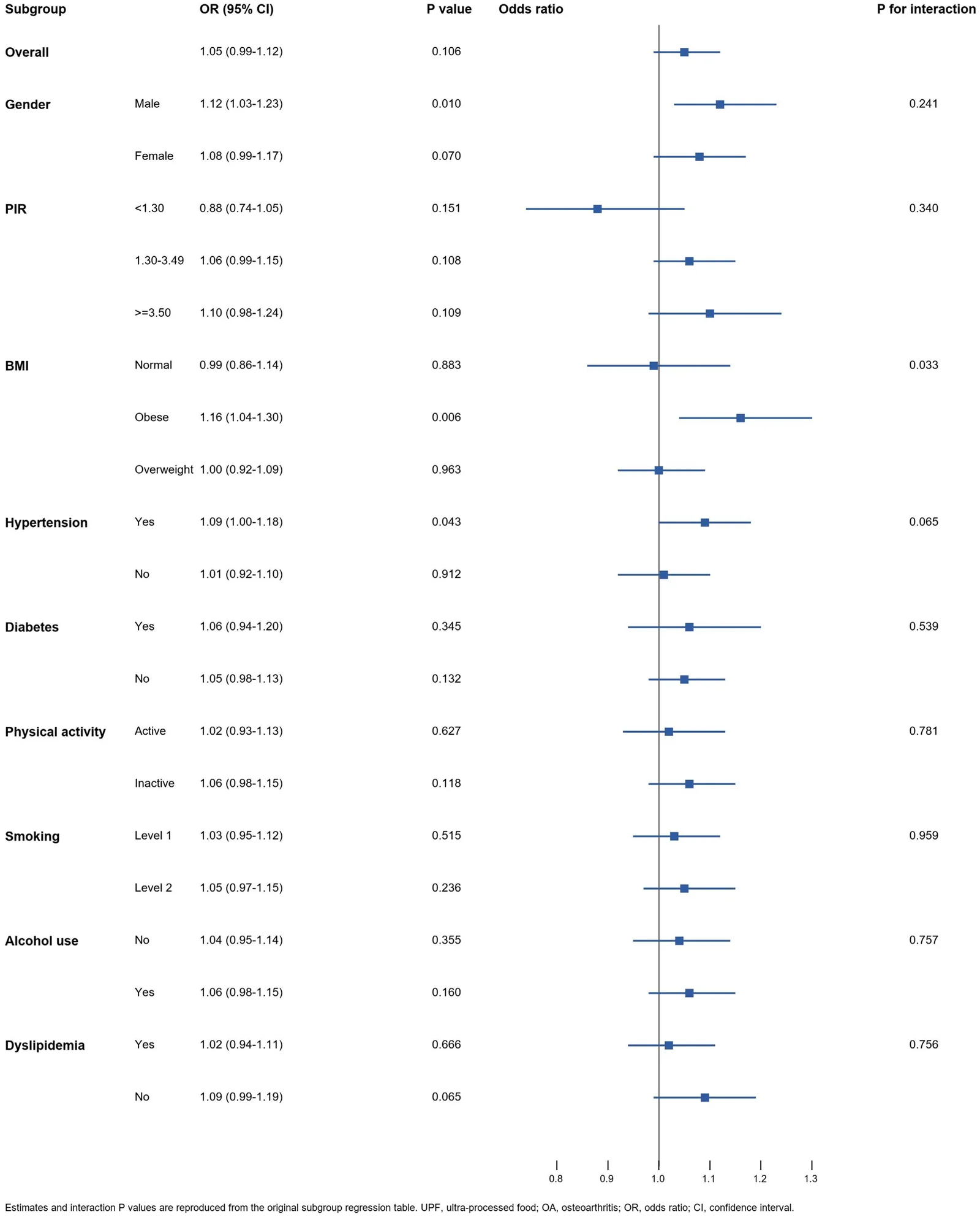
Subgroup analyses of the association between ultra-processed food intake and osteoarthritis prevalence. Forest plot displaying the odds ratios (ORs) and 95% confidence intervals for the association between UPF intake and osteoarthritis across various demographic and clinical subgroups. Subgroups are defined by poverty-income ratio (PIR), body mass index (BMI), hypertension, diabetes, physical activity, smoking status, alcohol consumption, dyslipidemia, and gender. The association was generally consistent across most subgroups, with numerically higher effect estimates observed in individuals with obesity (BMI ≥ 30 kg/m^2^) and hypertension, with a statistically significant interaction for BMI category (*P* for interaction = .033) but not for hypertension status (*P* = .065). UPF = ultra-processed food.

Notably, numerically higher effect estimates were observed among individuals with obesity (BMI ≥ 30 kg/m^2^: OR = 1.164, 95% CI: 1.046–1.295, *P* = .006), and among participants with hypertension (OR = 1.086, 95% CI: 1.003–1.175, *P* = .043). The interaction term for BMI group was statistically significant (*P* for interaction = .033), whereas the interaction term for hypertension status was not (*P* = .065), indicating heterogeneity across BMI categories but not hypertension status. No significant interaction was observed for other stratifying variables, including PIR, diabetes, PA, smoking, alcohol use, dyslipidemia, or gender.

### 3.5. LASSO regression analysis

To further refine variable selection and identify the most relevant predictors of osteoarthritis, a LASSO binomial regression model was constructed using 17 candidate variables, including demographic, clinical, and dietary factors. The model applied 10-fold cross-validation and minimized deviance as the performance metric.

The optimal penalization parameter was determined based on the minimum mean cross-validated error (lambda minutes = 0.0004), with a corresponding log lambda value of approximately–7.78 (Fig. 3A). As shown in the coefficient path plot (Fig. 3B), the magnitude of variable coefficients progressively shrank with increasing lambda values, with many predictors’ coefficients approaching zero. At the optimal lambda, the retained variables with nonzero coefficients included Gender (β = 0.7078), Age (β = 0.0499), BMI group (β = 0.2934), Education (β = 0.1228), PIR group (β = 0.1803), HDL (β = 0.0083), PA (β = 0.0682), Drink (β = 0.0469), HsC-reactive protein (β = 0.0055), and ultra-processed. kcal (β = 0.0002). Several variables demonstrated negative coefficients, including Hypertension (β = –0.3433), Smoke (β = –0.3425), Dyslipidemia (β = –0.3986), and Marital status (β = –0.1376), indicating a potential inverse relationship with OA risk.

**Figure 3.**
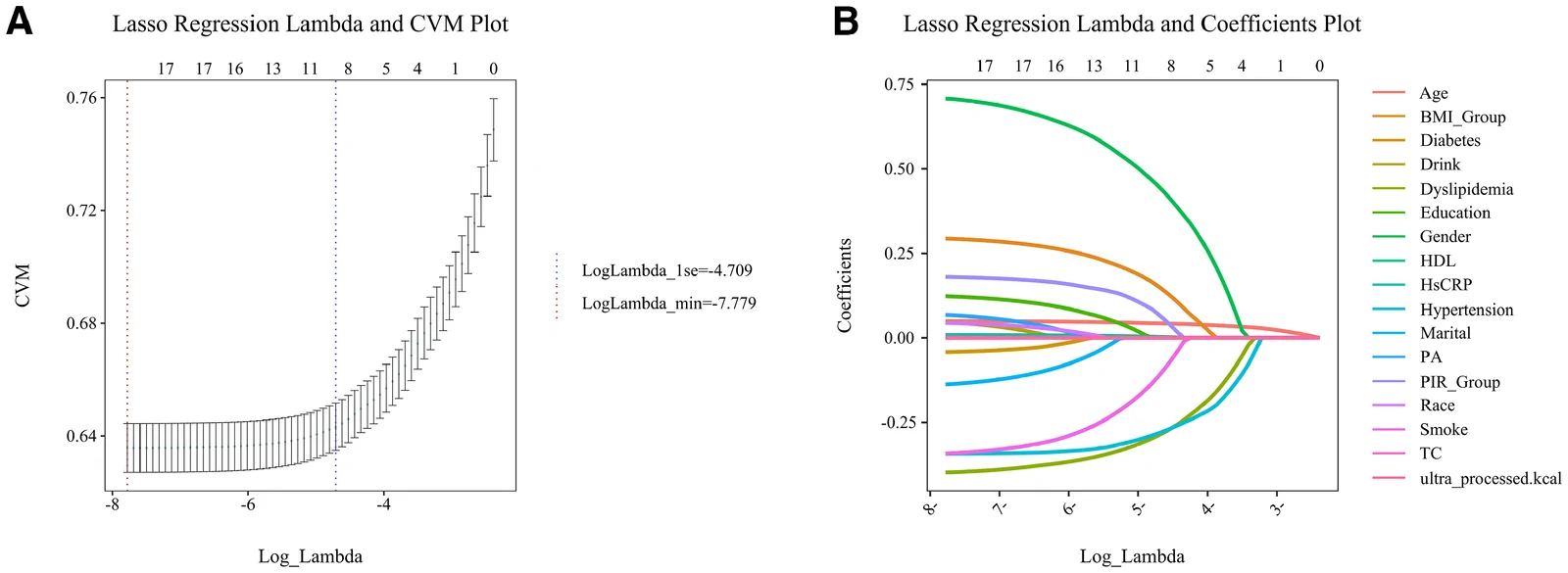
Feature selection using Lasso regression. (A) Ten-fold cross-validation curve for the Lasso binomial regression model. The Y-axis represents the binomial deviance, and the X-axis represents the log(Lambda) value. The left vertical dotted line indicates the lambda value at the minimum mean cross-validated error (lambda_min), and the right vertical line indicates the lambda value within 1 standard error of the minimum (lambda_1se). (B) Lasso coefficient paths showing the trajectories of variable coefficients as the penalty parameter (Lambda) increases. Each colored line represents a predictor variable. Variables with coefficients that shrink to zero at the selected lambda are excluded from the final model.

### 3.6. Performance of machine learning models

To comprehensively evaluate the performance and generalizability of various machine learning models in classifying OA, we compared 6 widely used algorithms: XGBoost, LightGBM, CatBoost, GBDT, Random Forest, and NB: across training, testing, and cross-validation datasets.

In the training set (Fig. 4), all models except NB demonstrated high predictive performance. XGBoost achieved an accuracy of 0.9603, F1-score of 0.9604, and AUROC of 0.9942. LightGBM and CatBoost also performed well with AUROCs of 0.9704 and 0.9654, respectively. GBDT reached perfect metrics (AUROC = 1.0), suggesting overfitting. In contrast, NB yielded substantially lower performance (AUROC = 0.6321, F1-score = 0.6445), indicating limited utility in this setting. In the testing set, model performance was more differentiated, with XGBoost maintaining the best classification performance (accuracy = 0.8841, F1-score = 0.8843, AUROC = 0.9553). LightGBM and CatBoost followed with AUROCs of 0.9390 and 0.9288, respectively. The ROC curves (Fig. 5A) visually confirmed XGBoost’s superior discrimination, while the calibration plot (Fig. 5B) showed it had the lowest calibration error (0.085), indicating high reliability of predicted probabilities.

**Figure 4.**
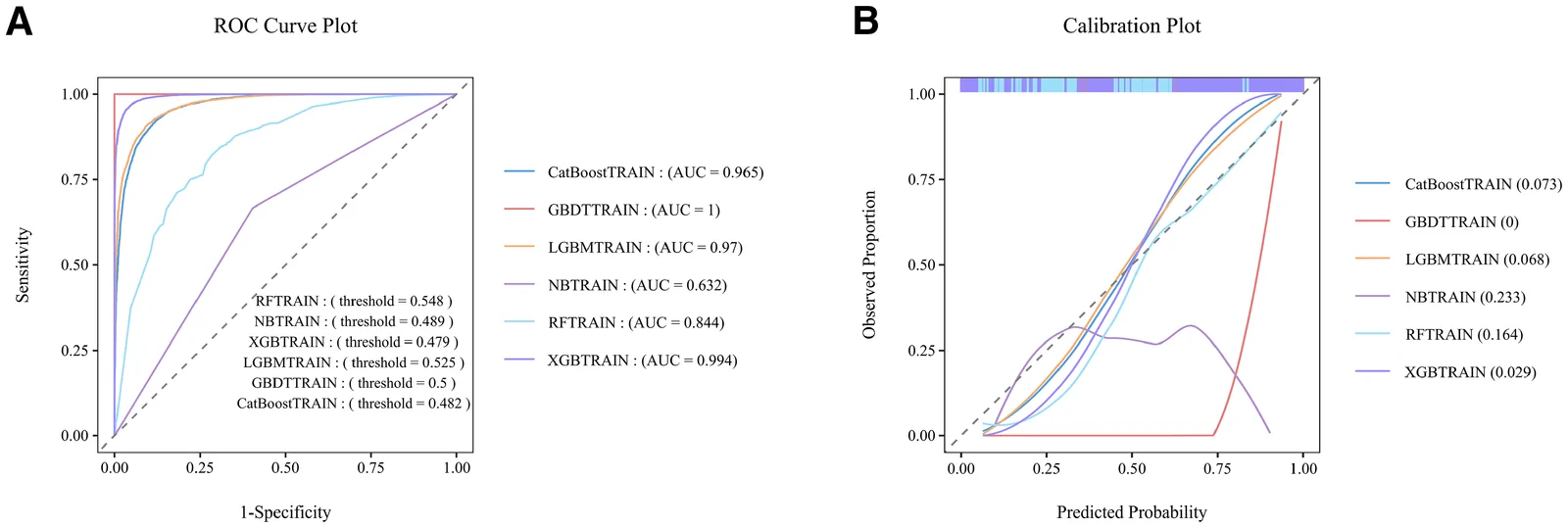
Performance evaluation of machine learning models on the training set. (A) Receiver Operating Characteristic (ROC) curves for the 6 machine learning models on the training dataset. The Area Under the ROC Curve (AUROC) values are shown in the legend, with the XGBoost model demonstrating near-perfect discrimination (AUROC = 0.994). (B) Calibration curves for the models on the training set. The dashed diagonal line represents ideal calibration. The XGBoost and GBDT models showed excellent calibration, while the Naïve Bayes model was poorly calibrated.

**Figure 5.**
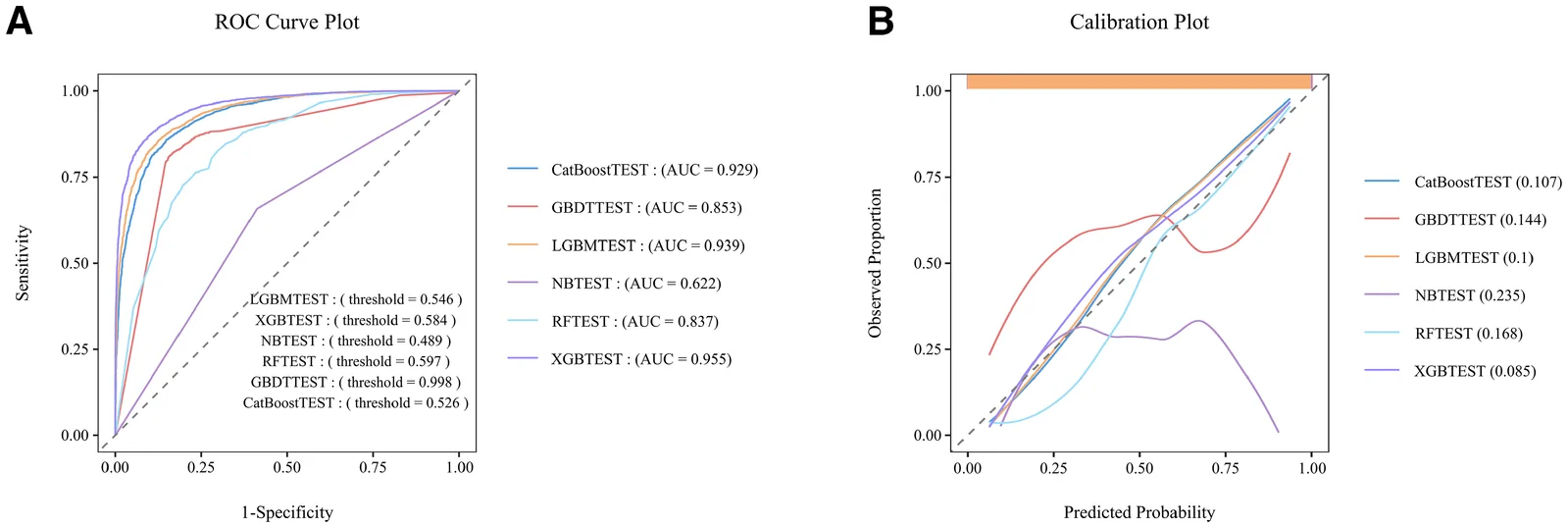
Performance evaluation of machine learning models on the testing set. (A) ROC curves for the 6 machine learning models on the independent testing dataset. The XGBoost model maintained the highest AUROC (0.955), indicating stronger discrimination in the held-out test set. (B) Calibration curves for the models on the testing set. The XGBoost model exhibited the lowest calibration error (0.085), indicating that its predicted probabilities were most reliable.

To assess model robustness and avoid overfitting bias, we performed 5-fold cross-validation using the XGBoost model only, given its best performance. Across the 5 validation folds, AUROC values remained consistently high, ranging from 0.959 to 0.964 (Fig. 6A), and calibration errors were also low and stable (range: 0.076–0.082, Figure 6B), indicating stable internal cross-validation performance and probability calibration.

**Figure 6.**
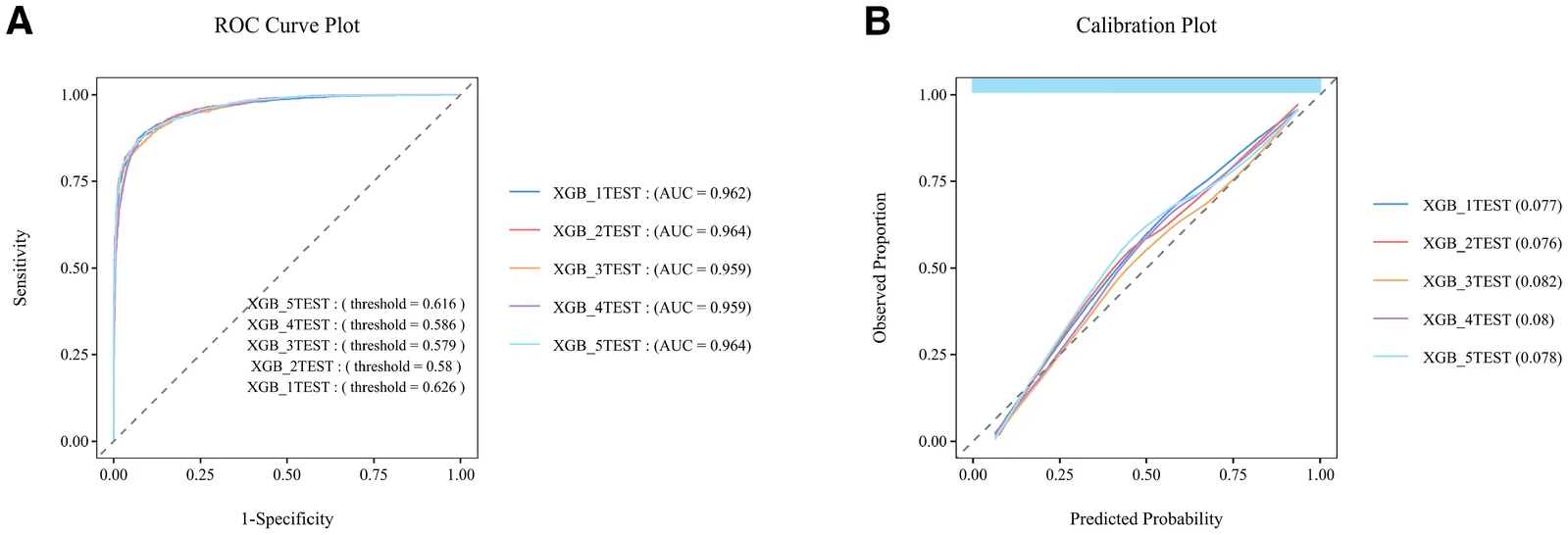
Robustness assessment of the XGBoost model using 5-fold cross-validation. (A) ROC curves from the 5 independent cross-validation folds. The consistently high and stable AUROC values (ranging from 0.959–0.964) across all folds demonstrate the model’s robustness and low variance. (B) Calibration curves for the corresponding 5 folds. The low and stable calibration errors (ranging from 0.076–0.082) indicate stable internal probability calibration across different data subsets.

### 3.7. Model interpretation using SHAP analysis

To interpret the predictions of the final XGBoost model, SHAP values were computed to quantify the contribution of each input variable to the model’s output. As shown in Figure 7A, the top features contributing to osteoarthritis prediction included age, BMI group, hypertension, HDL cholesterol, PA, and UPF intake. These variables had the highest average absolute SHAP values, indicating their strong influence on the model’s output probability.

**Figure 7.**
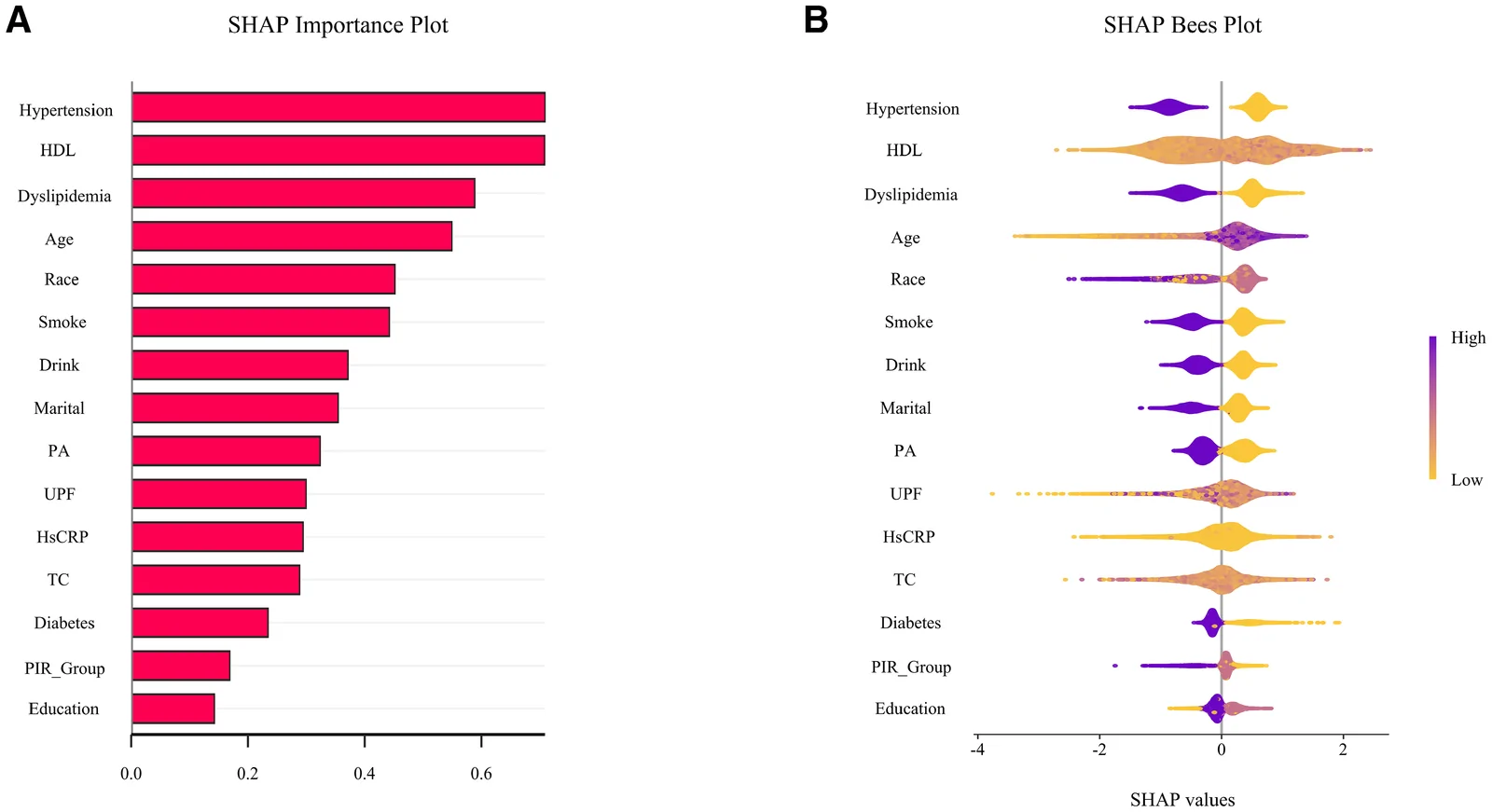
Interpretation of the XGBoost model using SHapley Additive exPlanations (SHAP). (A) Summary bar plot of feature importance, ranked by the mean absolute SHAP value. Features such as age, BMI group, and UPF intake were the most influential predictors of osteoarthritis in the model. (B) Beeswarm plot illustrating the distribution of SHAP values for the top features. Each point represents a single participant. The color indicates the feature value (red for high, blue for low), and the horizontal position indicates the signed contribution to the model output. This plot summarizes the direction and magnitude of each feature’s contribution within the model. BMI = body mass index, SHAP = Shapley Additive Explanations.

A SHAP beeswarm plot (Fig. 7B) further illustrated the distribution of SHAP values across individuals. For example, older age and higher BMI tended to have positive SHAP values, whereas HDL levels and PA had negative SHAP values within this model. Notably, the relationship between some variables and the predicted outcome was nonlinear and heterogeneous, as observed in the spread of SHAP values for UPF and PIR group.

These results suggest that the XGBoost model not only achieves high predictive performance, but also captures meaningful clinical patterns that align with known correlates of osteoarthritis. Additional Incremental Prediction Analysis. The 70:30 split yielded 8615 training participants and 3692 test participants, with OA prevalences of 12.39% and 12.38%, respectively. In the test set (457 OA events), AUROC was 0.909 for the conventional model and 0.959 after adding UPF; the difference was 0.050 (95% CI: 0.022–0.081; paired DeLong *P* = .018). The split and incremental-performance summaries are provided in Supplemental Digital Content 9 and 10 (Tables S8 and S9, Supplemental Digital Content 7); the underlying paired DeLong and test-set performance outputs are provided in Supplemental Digital Content 13 and 14 (Files 3 and 4, Supplemental Digital Content 13 and 14).

## 4. Discussion

In this cross-sectional analysis of the NHANES 2015 to 2020 dataset, we found that UPF consumption was associated with OA prevalence. In crude models, higher UPF intake appeared to be protective, as participants with OA were older, had more comorbidities, and reported lower UPF consumption. However, after adjusting for potential confounders, including age, sex, race, socioeconomic status, lifestyle factors, BMI, and comorbidities, the association reversed. Individuals in the highest quartile of UPF intake had approximately 24% higher odds of self-reported OA compared to those in the lowest quartile. RCS analysis revealed a linear dose-response relationship. Subgroup analysis indicated heterogeneity by BMI category (*P* for interaction = .033), with a statistically significant association in the obesity subgroup. Furthermore, exploratory machine-learning analyses assigned UPF intake a nonzero contribution to prediction alongside age, BMI, hypertension, high-density lipoprotein cholesterol, and PA. The best-performing model achieved an area under the receiver operating characteristic curve (AUC) of 0.96 in cross-validation. Although participants with OA had a higher mean PIR, they were less likely to fall into the lowest PIR category (<1.30). These findings are not contradictory because the mean PIR reflects the overall distribution, whereas the categorical comparison specifically captures the lower-income group. The higher proportion of participants with OA in the highest PIR category (≥3.50) may have contributed to their higher mean PIR.

These findings contribute to a deeper understanding of modifiable drivers of OA within the context of a disorder whose global burden continues to rise.^[17,18]^ Recent Global Burden of Disease estimates indicate that OA affected over 600 million people worldwide in 2021, with incident cases and disability-adjusted life years steadily increasing over the past 3 decades.^[17]^ Women have been disproportionately affected, and high BMI has been strongly implicated as a major contributor.^[19]^ The higher proportion of women with OA observed in our study is consistent with established sex differences in OA epidemiology and may partly reflect postmenopausal hormonal changes, as well as sex-related differences in joint anatomy, biomechanics, and body composition. The observation that intake of UPF is positively associated with OA prevalence builds upon this epidemiological backdrop, suggesting that food processing itself may be a key factor in the obesity-OA nexus. Numerous observational studies have established links between diets rich in UPFs and weight gain, metabolic syndrome, and chronic inflammatory conditions. These associations are attributed to the high energy density, refined carbohydrates, sodium, and additives present in UPFs, as well as their lack of fiber, protein, and micronutrients.^[10]^ A projection study has estimated that OA incidence will nearly double by 2050,^[19]^ underscoring the long-term risks associated with the consumption of UPFs,^[10]^ while highlighting the inverse associations between dietary fiber intake and OA risk in reducing inflammatory responses.^[20]^ A scoping review of nineteen studies has also shown that UPF consumption is associated with lower bone mineral density and an increased risk of osteoporosis and knee OA.^[21]^ Our findings are consistent with these observations, extending the association to a large, racially diverse U.S. adult population. Importantly, our analysis demonstrates a graded relationship between UPF intake and OA prevalence, after adjustment for the specified covariates. In contrast to the OA Initiative, which reported that higher UPF consumption was associated with more severe pain, functional limitations, and thinner cartilage, particularly among women,^[22]^ our analysis found a consistent association across both sexes. The interaction analysis identified statistically significant heterogeneity across BMI categories but not across hypertension status. These differences may reflect variations in population characteristics, methods of measuring UPF intake, or the endpoints examined (structural versus symptomatic).

The observed linear pattern should be evaluated in prospective studies with repeated dietary assessment and objective OA outcomes. This finding aligns with existing evidence indicating that dietary patterns rich in unprocessed plant foods confer musculoskeletal benefits. In NHANES-based analyses, higher intakes of dietary fiber, folate, and niacin were associated with 27 to 33% lower odds of OA, whereas protein exhibited a J-shaped relationship.^[23]^ A separate cross-sectional analysis of NHANES data found that participants in the highest quartile of fiber intake had a 27% lower risk of OA compared to those in the lowest quartile.^[20]^ Furthermore, high total or grain fiber intake in prospective cohorts was predictive of a lower likelihood of developing moderate or severe knee pain over an eight-year period.^[24]^ In contrast, the present study focused on composite UPF intake rather than specific nutrients, but these studies evaluate exposures that differ from the composite UPF measure used here and do not establish effects of dietary change on OA. Mechanistic hypotheses linking UPFs to OA propose that emulsifiers and additives compromise gut mucosal integrity,^[11,12]^ elevate circulating lipopolysaccharides,^[13]^ and trigger activation of synovial toll-like receptor 4 and the NLRP3 inflammasome.^[4,7]^ Additionally, excessive intake of AGEs may contribute to cartilage stiffness and activate the receptor for AGEs (RAGE) signaling.^[5,14]^ Experimental evidence from a rat model indicates that a high-fat/high-sucrose diet can induce osteoarthritis-like degeneration in the knee and shoulder joints.^[25]^ Thus, our findings integrate nutritional epidemiology with pathophysiological insights, suggesting that cumulative exposure to processed food constituents, rather than isolated nutrients, may contribute to chronic low-grade inflammation and metabolic stress: key features of OA.^[26]^

The subgroup analyses further emphasize the complex interplay between UPFs and metabolic risk factors. We observed that the association between UPF intake and OA was stronger among individuals with obesity (BMI ≥ 30 kg/m^2^) and those with hypertension, and the BMI interaction was statistically significant. This pattern is biologically plausible: obesity promotes synovitis, inflammation of the infrapatellar fat pad, and adipokine secretion: processes that accelerate cartilage degradation and pain.^[26]^ The Global Burden of Disease analysis identified high BMI as the most significant modifiable risk factor for OA,^[19]^ and diets rich in UPFs are major contributors to obesity and visceral adiposity.^[15]^ Dietary interventions that reduce caloric intake and promote weight loss have consistently led to improvements in pain and function in OA. A recent meta-analysis found that reduced-energy diets resulted in the most substantial improvements in pain and function, whereas Mediterranean diets showed more modest effects.^[27]^ These cross-sectional findings do not establish that dietary modification prevents OA. These results align with case-control studies that have shown that diets with high dietary acid load^[28]^ or low phytochemical index significantly increase the odds of knee OA.^[29]^ In parallel, adherence to the Mediterranean diet: characterized by high consumption of fruits, vegetables, whole grains, olive oil, and fish: has been associated with reduced OA risk, improved pain scores, and better quality of life.^[30]^ Prospective studies are needed to determine whether changes in dietary patterns influence OA outcomes.

Although cross-sectional data preclude causal inference, evidence from prospective and genetic studies provides context but does not establish causal effects of UPF intake on OA. A prospective cohort study found that a higher Dietary Inflammatory Index: a score reflecting the pro-inflammatory potential of the diet: was associated with an increased incidence of radiographic and symptomatic knee OA, partly mediated by BMI.^[31]^ Two Mendelian randomization analyses have also implicated diet in OA risk: one study found that genetically predicted higher consumption of coffee and peas increased OA risk, whereas greater intake of watercress and cheese appeared protective^[32]^;another reported that genetically elevated coffee intake increased knee OA risk, while higher consumption of dried fruits, grains, cheese, and oily fish was protective.^[33]^ Although UPF consumption cannot be directly inferred from these genetic instruments, these findings suggest that specific food components may have causal effects on OA and underscore the need for further genetic and metabolic studies to better understand the contributions of UPF constituents to OA pathogenesis. Future prospective cohort studies with repeated dietary assessments and objective OA outcomes (radiographic or MRI-based) are essential to validate these associations and to test whether reductions in UPF intake can reduce incident OA, independent of weight change. Although machine learning models showed high discrimination, these findings should be interpreted as exploratory prediction rather than clinical prediction tools because models were developed and internally evaluated within the same NHANES dataset without external validation.

Machine-learning analysis provided exploratory information on multivariable prediction patterns. The XGBoost model assigned high predictive contribution to age, BMI, hypertension, PA, high-density lipoprotein cholesterol, and UPF intake. These model-based contributions do not establish independent clinical determinants or causal effects. The model showed high internal discrimination and calibration, but requires external validation before any clinical application. However, caution is warranted: the model was trained and tested within the same survey dataset, and external validation in independent cohorts is necessary. Furthermore, while Shapley additive explanations were employed to enhance interpretability, machine-learning models remain inherently challenging to interpret. The incremental predictive value of UPF intake, relative to simpler models, requires further investigation. The additional comparison with and without UPF showed improved internal test-set discrimination after adding UPF.

## 5. Limitations

This study has several limitations. First, the cross-sectional design precludes causal inference, as reverse causality cannot be ruled out: individuals with established OA may modify dietary behaviors because of chronic pain, reduced mobility, functional limitations, or healthcare advice. Such reverse dietary changes may influence the observed association between UPF intake and OA prevalence. Prospective studies are needed to establish the temporal relationship between UPF consumption and OA onset. Second, dietary intake was assessed using a single 24-hour recall, which may not accurately reflect habitual dietary patterns because of substantial within-person variability and potential misreporting or recall bias. Consequently, UPF exposure may have been misclassified, which could influence the magnitude of the observed association. Repeated dietary assessments or longitudinal dietary measurements would provide a more accurate evaluation of habitual UPF intake. NHANES does not provide an official NOVA classification or complete brand-level ingredient information; residual UPF misclassification cannot be excluded. In addition, the archived RCS record retained the transformed scale and reference value but not the exact transformation formula.

In addition, different approaches for quantifying UPF exposure, such as the percentage contribution of UPF to total energy intake or total food weight, may reflect different dimensions of dietary exposure. Future studies using standardized relative UPF metrics are needed to further validate these findings. Third, OA status was self-reported without radiographic confirmation, raising the possibility of misclassification. However, any such misclassification would likely bias the associations toward the null. Fourth, residual confounding remains a concern despite adjustment for numerous covariates, particularly socioeconomic factors, comorbidities, and other dietary variables. Fifth, site-specific OA (e.g., knee, hip, hand) was not examined, nor were objective measures of inflammatory biomarkers, gut microbiota, or AGE incorporated. Such mechanistic data would provide valuable insights into the pathways linking UPFs to joint degeneration. Finally, the machine-learning models may be prone to overfitting within the NHANES sample, and their performance may vary when applied to other populations or when predicting incident, rather than prevalent, OA.

## 6. Conclusion

In conclusion, this cross-sectional study based on NHANES (2015–2020) found positive associations between UPF intake and OA in the U.S. population after adjusting for potential confounders. The group with the highest intake of UPFs had approximately 24% higher odds of OA than that of the group with the lowest intake, demonstrating a clear linear dose-response relationship. The interaction analysis identified significant heterogeneity across BMI categories (*P* for interaction = .033), whereas the hypertension interaction was not statistically significant (*P* = .065). Machine-learning analyses further identified age, body mass index (BMI) and UPF intake as the most influential predictors, indicating that dietary processing may show different associations with OA prevalence across metabolic profiles. These findings describe associations and do not establish that dietary modification prevents OA. Further research and randomized controlled trials, along with research on mechanisms such as the gut-joint axis, are needed to confirm these results, and determine effective dietary strategies for OA prevention and management.

## Acknowledgments

This study received no specific funding from any public, commercial, or not-for-profit organizations. The authors declare no conflict of interest.

## Author contributions

**Conceptualization:** Hongming Liang.

**Formal analysis:** Hongming Liang, Xin Liu.

**Methodology:** Hongming Liang, Cheng Li.

**Writing – review & editing:** Hongming Liang, Hongchun Peng, Li Shen, Xin Liu, Cheng Li, Liwen Xue.

**Data curation:** Hongchun Peng.

**Investigation:** Hongchun Peng.

**Project administration:** Li Shen.

**Supervision:** Li Shen.

**Validation:** Liwen Xue.




























