# Supplementary material for: Association between ultra-processed food intake and osteoarthritis prevalence among U.S. adults: A cross-sectional study using NHANES data
Source: Medicine (Baltimore). 2026 Oct 30;105(44):e51343. doi: 10.1097/MD.0000000000051343 (PMC13727231; doi:10.1097/MD.0000000000051343)
Supplement: Supplementary file 1 [file medi-105-e51343-s001.docx]

**Supplemental Digital Content 1, Table S1. Participant Selection**

| **stage** | **n** |
| --- | --- |
| Combined participant data | 25531 |
| Age >=40 years | 14951 |
| Reliable Day 1 recall with dietary weight | 14018 |
| Known OA outcome and nonmissing UPF | 12723 |
| Primary-model complete cases | 12307 |

n, unweighted number of participants; OA, osteoarthritis; UPF, ultra-processed food.
