# Supplementary material for: Association between ultra-processed food intake and osteoarthritis prevalence among U.S. adults: A cross-sectional study using NHANES data
Source: Medicine (Baltimore). 2026 Oct 30;105(44):e51343. doi: 10.1097/MD.0000000000051343 (PMC13727231; doi:10.1097/MD.0000000000051343)
Supplement: Supplementary file 2 [file medi-105-e51343-s002.docx]

**Supplemental Digital Content 5, Table S4b. NOVA Classification Rule Audit: Intake Contribution**

| **Cycle** | **Classification category** | **Food codes** | **Total kcal** | **Total grams** | **Energy share (%)** | **Food-weight share (%)** |
| --- | --- | --- | --- | --- | --- | --- |
| 2015-2016 | WWEIA category rule | 1,570 | 6,561,511 | 4,295,547 | 40.31 | 18.55 |
| 2015-2016 | food-description rule | 285 | 1,059,132 | 875,265 | 6.51 | 3.78 |
| 2015-2016 | industrial-source/description rule | 299 | 710,896 | 1,106,979 | 4.37 | 4.78 |
| 2015-2016 | Not group 4 | 436 | 1,121,989 | 9,274,400 | 6.89 | 40.06 |
| 2015-2016 | Not group 4 | 169 | 844,866 | 626,638 | 5.19 | 2.71 |
| 2015-2016 | Not group 4 | 2,326 | 5,980,588 | 6,974,255 | 36.74 | 30.12 |
| 2017-2020 | WWEIA category rule | 1,713 | 10,195,297 | 6,525,704 | 41.07 | 18.63 |
| 2017-2020 | food-description rule | 272 | 1,564,097 | 1,308,117 | 6.30 | 3.74 |
| 2017-2020 | industrial-source/description rule | 322 | 1,032,280 | 1,532,482 | 4.16 | 4.38 |
| 2017-2020 | Not group 4 | 456 | 1,943,786 | 14,421,247 | 7.83 | 41.18 |
| 2017-2020 | Not group 4 | 163 | 1,310,116 | 904,944 | 5.28 | 2.58 |
| 2017-2020 | Not group 4 | 2,355 | 8,776,646 | 10,328,806 | 35.36 | 29.49 |

kcal, kilocalories; energy share and food-weight share are percentages within each survey cycle.
