# Supplementary material for: Association between ultra-processed food intake and osteoarthritis prevalence among U.S. adults: A cross-sectional study using NHANES data
Source: Medicine (Baltimore). 2026 Oct 30;105(44):e51343. doi: 10.1097/MD.0000000000051343 (PMC13727231; doi:10.1097/MD.0000000000051343)
Supplement: Supplementary file 3 [file medi-105-e51343-s004.docx]

**Supplemental Digital Content 3, Table S3. Osteoarthritis Outcome Response-Path Audit**

| **cycle** | **MCQ160A** | **MCQ195** | **oa_status** | **n** |
| --- | --- | --- | --- | --- |
| 2015_2016 | 1 | 1 | OA | 616 |
| 2015_2016 | 1 | 2 | no OA | 292 |
| 2015_2016 | 1 | 3 | no OA | 18 |
| 2015_2016 | 1 | 4 | no OA | 142 |
| 2015_2016 | 1 | 9 | no OA | 402 |
| 2015_2016 | 2 | NA | no OA | 4238 |
| 2015_2016 | 9 | NA | no OA | 11 |
| 2015_2016 | NA | NA | unknown | 3856 |
| 2017_2020 | 1 | 1 | OA | 1182 |
| 2017_2020 | 1 | 2 | no OA | 574 |
| 2017_2020 | 1 | 3 | no OA | 45 |
| 2017_2020 | 1 | 4 | no OA | 316 |
| 2017_2020 | 1 | 7 | no OA | 3 |
| 2017_2020 | 1 | 9 | no OA | 692 |
| 2017_2020 | 2 | NA | no OA | 6394 |
| 2017_2020 | 9 | NA | no OA | 26 |
| 2017_2020 | NA | NA | unknown | 5754 |

OA, osteoarthritis; NA, missing or not applicable questionnaire response; n, unweighted number of participants. MCQ160A records the physician-diagnosed arthritis response; MCQ195 records the reported arthritis type.
