# Supplementary material for: Association between ultra-processed food intake and osteoarthritis prevalence among U.S. adults: A cross-sectional study using NHANES data
Source: Medicine (Baltimore). 2026 Oct 30;105(44):e51343. doi: 10.1097/MD.0000000000051343 (PMC13727231; doi:10.1097/MD.0000000000051343)
Supplement: Supplementary file 4 [file medi-105-e51343-s005.docx]

**Supplemental Digital Content 2, Table S2. Variable Coding Dictionary**

| **construct** | **source_variable_or_algorithm** | **coding** |
| --- | --- | --- |
| Osteoarthritis | MCQ195 = 1; otherwise 0 when MCQ160A is observed | Self-reported physician diagnosis; unknown values excluded |
| Hypertension | BPQ020 = 1 | Yes vs no; unknown/refused excluded |
| Diabetes | DIQ010 = 1 | Yes vs no; unknown/refused excluded |
| High cholesterol | BPQ080 = 1 | Yes vs no; unknown/refused excluded |
| Smoking | SMQ020/SMQ040 | Never, former, current; unknown/refused excluded |
| Education | DMDEDUC2: 1-2, 3, 4-5 | Less than high school, high school/GED, more than high school |
| Physical activity | PAQ/PAD work and recreation minutes; >=150 moderate-equivalent min/week | Binary active/not active; incomplete activity responses excluded |
| Poverty-income ratio | INDFMPIR; descriptive categories <1.30, 1.30-3.49, >=3.50 | Continuous in models; categories only for descriptive reporting |
| Dietary weight | WTDRD1 x 2/5.2 or WTDRD1PP x 3.2/5.2 | Day-1 dietary weights for combined 5.2-year analytic period |

OA, osteoarthritis; GED, General Educational Development; NHANES, National Health and Nutrition Examination Survey. Questionnaire variable codes and dietary-weight variables are defined in the table.
