# Supplementary material for: Association between ultra-processed food intake and osteoarthritis prevalence among U.S. adults: A cross-sectional study using NHANES data
Source: Medicine (Baltimore). 2026 Oct 30;105(44):e51343. doi: 10.1097/MD.0000000000051343 (PMC13727231; doi:10.1097/MD.0000000000051343)
Supplement: Supplementary file 5 [file medi-105-e51343-s006.docx]

**Supplemental Digital Content 7, Table S6. Relative UPF Exposure Sensitivity Analyses**

| **Exposure** | **OR (95% CI)** | **P value** |
| --- | --- | --- |
| Absolute UPF intake per 100 g/day | 1.01 (0.99-1.03) | 0.141 |
| UPF energy percentage per 10 percentage points | 1.20 (1.02-1.40) | 0.025 |
| UPF food-weight percentage per 10 percentage points | 1.20 (1.04-1.40) | 0.017 |

UPF, ultra-processed food; OR, odds ratio; CI, confidence interval. Estimates are from the fully adjusted survey-weighted logistic regression model.
