# Supplementary material for: Association between ultra-processed food intake and osteoarthritis prevalence among U.S. adults: A cross-sectional study using NHANES data
Source: Medicine (Baltimore). 2026 Oct 30;105(44):e51343. doi: 10.1097/MD.0000000000051343 (PMC13727231; doi:10.1097/MD.0000000000051343)
Supplement: Supplementary file 6 [file medi-105-e51343-s007.docx]

**Supplemental Digital Content 9, Table S8. Training and Test Split**

| **Subset** | **Participants** | **OA prevalence (%)** |
| --- | --- | --- |
| Training | 8,615 | 12.39 |
| Test | 3,692 | 12.38 |

OA, osteoarthritis. The training and test subsets comprise 70% and 30% of the analytical sample, respectively.
