# Supplementary material for: Association between ultra-processed food intake and osteoarthritis prevalence among U.S. adults: A cross-sectional study using NHANES data
Source: Medicine (Baltimore). 2026 Oct 30;105(44):e51343. doi: 10.1097/MD.0000000000051343 (PMC13727231; doi:10.1097/MD.0000000000051343)
Supplement: Supplementary file 7 [file medi-105-e51343-s008.docx]

**Supplemental Digital Content 4, Table S4a. NOVA Classification Rule Audit: Classification and Counts**

| **Cycle** | **Classification basis** | **Review flag** | **UPF** | **Food codes** | **Recall records** |
| --- | --- | --- | --- | --- | --- |
| 2015-2016 | WWEIA category rule | Rule positive | Yes | 1,570 | 27,622 |
| 2015-2016 | food description rule | Rule positive | Yes | 285 | 6,439 |
| 2015-2016 | industrial source/description rule | Rule positive | Yes | 299 | 6,756 |
| 2015-2016 | not classified as group 4 by prespecified rule | Clearly minimally processed description | No | 436 | 29,519 |
| 2015-2016 | not classified as group 4 by prespecified rule | Homemade or scratch description | No | 169 | 3,030 |
| 2015-2016 | not classified as group 4 by prespecified rule | Requires description review | No | 2,326 | 48,115 |
| 2017-2020 | WWEIA category rule | Rule positive | Yes | 1,713 | 41,709 |
| 2017-2020 | food description rule | Rule positive | Yes | 272 | 8,342 |
| 2017-2020 | industrial source/description rule | Rule positive | Yes | 322 | 9,116 |
| 2017-2020 | not classified as group 4 by prespecified rule | Clearly minimally processed description | No | 456 | 45,390 |
| 2017-2020 | not classified as group 4 by prespecified rule | Homemade or scratch description | No | 163 | 4,305 |
| 2017-2020 | not classified as group 4 by prespecified rule | Requires description review | No | 2,355 | 75,048 |

UPF, ultra-processed food; WWEIA, What We Eat in America. Recall records are food-intake records, not unique participants.
