# Supplementary material for: Association between ultra-processed food intake and osteoarthritis prevalence among U.S. adults: A cross-sectional study using NHANES data
Source: Medicine (Baltimore). 2026 Oct 30;105(44):e51343. doi: 10.1097/MD.0000000000051343 (PMC13727231; doi:10.1097/MD.0000000000051343)
Supplement: Supplementary file 8 [file medi-105-e51343-s009.docx]

**Supplemental Digital Content 6, Table S5. NOVA Classification Uncertainty Summary**

| **Metric** | **Value** |
| --- | --- |
| Participants | 20,715 |
| Mean rule-positive energy (%) | 49.54 |
| Median rule-positive energy (%) | 49.98 |
| Mean energy requiring description review (%) | 37.12 |
| Median energy requiring description review (%) | 34.67 |

Rule-positive energy is energy assigned as ultra-processed food by the prespecified NOVA rules. Energy requiring description review is the energy contribution from records flagged for further description review.
