# Supplementary material for: Association between ultra-processed food intake and osteoarthritis prevalence among U.S. adults: A cross-sectional study using NHANES data
Source: Medicine (Baltimore). 2026 Oct 30;105(44):e51343. doi: 10.1097/MD.0000000000051343 (PMC13727231; doi:10.1097/MD.0000000000051343)
Supplement: Supplementary file 9 [file medi-105-e51343-s010.docx]

**Supplemental Digital Content 8, Table S7. Model-Fit Statistics**

| **Metric** | **Value** |
| --- | --- |
| Minimum age | 40 |
| Analytic sample | 12,307 |
| OA cases | 1,524 |
| Survey design degrees of freedom | 40 |
| Cox-Snell pseudo-R2 | 0.131 |
| Nagelkerke pseudo-R2 | 0.236 |
| Weighted Brier score | 0.103 |
| Weighted Brier score 95% CI | 0.094-0.112 |
| Design-based decile calibration P value | 0.384 |

OA, osteoarthritis; CI, confidence interval; R², pseudo-R-squared. Statistics describe the primary survey-weighted logistic regression model.
