# Supplementary material for: Association between ultra-processed food intake and osteoarthritis prevalence among U.S. adults: A cross-sectional study using NHANES data
Source: Medicine (Baltimore). 2026 Oct 30;105(44):e51343. doi: 10.1097/MD.0000000000051343 (PMC13727231; doi:10.1097/MD.0000000000051343)
Supplement: Supplementary file 10 [file medi-105-e51343-s011.docx]

**Supplemental Digital Content 10, Table S9. Incremental Prediction Performance**

| **Comparison** | **AUROC or difference** | **95% CI** | **Paired DeLong P value** |
| --- | --- | --- | --- |
| Conventional model | 0.909 | 0.877-0.921 | - |
| Conventional model plus UPF | 0.959 | 0.928-0.971 | - |
| AUROC difference | 0.050 | 0.021-0.082 | 0.018 |

AUROC, area under the receiver operating characteristic curve; CI, confidence interval; UPF, ultra-processed food. The difference is the AUROC of the model including UPF minus that of the conventional model.
